# The late chromatoid body component TSSK2 is involved in translational regulation in elongating spermatids in mice

**DOI:** 10.1530/REP-25-0297

**Published:** 2025-10-15

**Authors:** Mari S Lehti, Lin Ma, Salli Kärnä, Samuli Laasanen, Ammar Ahmedani, Opeyemi Olotu, Matthieu Bourgery, Panyi Tran, Anu Sironen, Noora Kotaja

**Affiliations:** ^1^ Institute of Biomedicine, Integrative Physiology and Pharmacology Unit, University of Turku, Turku, Finland; ^2^ Centre for Population Health Research, Turku University Hospital and University of Turku, Turku, Finland; ^3^ Great Ormond Street Institute of Child Health, University College London, London, United Kingdom; ^4^ Natural Resources Institute Finland (Luke), Helsinki, Finland

**Keywords:** spermatogenesis, male fertility, elongating spermatids, RNP granules, translation, chromatoid body, TSSK2

## Abstract

**In brief:**

Temporally regulated translation is critical for late steps of spermatogenesis due to transcriptional silencing during chromatin condensation. This study shows that the function of the cytoplasmic granule, the late chromatoid body, is connected to translational regulation in condensing spermatids.

**Abstract:**

Spermatogenesis culminates in a dramatic morphological transformation, including a tight compaction of the chromatin and nuclear reshaping that largely silences transcription. Due to transcriptional silencing, the production of sperm-specific proteins needed for the morphological transformation requires active storage and translational regulation of mRNAs transcribed in earlier cell types. The germline-specific ribonucleoprotein (RNP) granule, the chromatoid body (CB), accumulates RNAs and has a role in RNA regulation in early haploid cells (round spermatids). In late haploid cells (elongating spermatids), the CB is transformed into the so-called late-CB, whose function in RNA regulation has remained elusive. Here we characterized the function of the late-CB by identifying proteins and RNAs interacting with the known late-CB marker, testis-specific serine/threonine-protein kinase 2 (TSSK2). We showed that TSSK2 and the late-CB associate with translation initiation factors and ribosomal proteins. Furthermore, we revealed an association of TSSK2 with a specific set of mRNAs that are enriched in polysome fractions in elongating spermatids, supporting the role of the late-CB in temporally regulated translation. These results link the function of the late-CB to RNA regulation during late spermatogenesis for the first time, providing important novel information about the RNA regulatory processes required for spermatogenesis and male fertility.

## Introduction

Spermatogenesis, taking place in the seminiferous tubules of the testis, includes massive morphological changes that transform haploid round spermatids into mature spermatozoa. During this post-meiotic haploid differentiation (spermiogenesis), the sperm head undergoes tight chromatin compaction and acquires its typical shape, and sperm-specific structures, such as the acrosome and the flagellum, are constructed. Spermiogenesis in mice is divided into 16 steps. During the first eight steps (steps 1–8), haploid cells retain the round nuclear shape (round spermatids), and the morphological transformation takes place during steps 9–16 (elongating spermatids). Finally, spermatogenesis is finalized by discarding all unnecessary proteins and cell organelles in the residual body when mature sperm are released from the seminiferous epithelium into the lumen of the seminiferous tubule.

Chromatin condensation takes place from step 11 onwards, when histones are replaced first by transition proteins and then by protamines. Tight compaction of the chromatin by protamines prohibits transcription for several days before the completion of spermiogenesis. Therefore, sperm development requires temporal regulation of protein translation, and the necessary genes are transcribed well in advance, with the transcripts stored in ribonucleoprotein (RNP) complexes in a translationally repressed state before their controlled and stage-specific translation (Kleene *et al.* 1984, Mali *et al.* 1989, Steger 1999, Kleene 2003). During the time of high transcriptional activity, the male germ cell-specific RNP granule, the chromatoid body (CB), appears in the cytoplasm of round spermatids, where it accumulates RNA and a variety of RNA-binding proteins (Lehtiniemi & Kotaja 2018). Based on its protein and RNA composition, the CB has been suggested to be involved in the regulation of mRNAs that are translated later in elongating spermatids.

During mid-spermiogenesis at the onset of spermatid elongation (steps 8–9), the CB decreases in size and is transformed into a specific late-CB structure, although the exact structural and functional relationship between the CB and the late-CB is still unclear. The CB and late-CB morphological characterization was described already in 1970 when Fawcett and colleagues presented impressive ultrastructural imaging of the late-CB during the late steps of spermiogenesis (Fawcett *et al.* 1970). The late-CB contains two parts: the satellite, which is located in the cytoplasm, and the ring, which is formed around the sperm tail close to the basal body. As spermiogenesis proceeds, the ring moves in close association with the septin-based annulus along the sperm tail, making room for the mitochondrial sheath to form (Kwitny *et al.* 2010, Shang *et al.* 2010).

**Figure 1:**
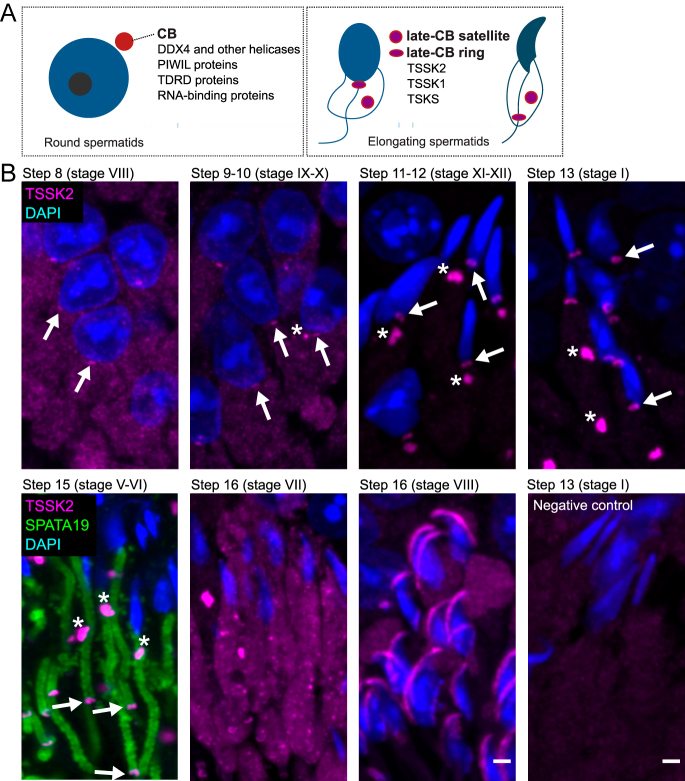
Localization of TSSK2 during late spermiogenesis. (A) Schematic drawing illustrates the CB and its main components in round spermatids, and the late-CB satellite and ring containing TSKS, TSSK1, and TSSK2 in elongating spermatids. (B) Immunofluorescence on adult mouse testis sections with anti-TSSK2 antibody. TSSK2 signal (magenta) appears as a small granule (arrow) in step 8 spermatids before it divides into a ring (arrow) and a satellite (asterisk) during the following steps. In step 15 spermatids, mitochondria (SPATA19, green) are aligned in the midpiece of the tail, and the late-CB ring has traveled along the sperm tail to the border between the midpiece and the principal piece. Just before spermiation, in step 16 spermatids at stage VIII, the TSSK2 signal is detected close to the acrosomal area. DAPI (blue) stains the nuclei. Scale bar 2 μm.

The molecular composition and function of the late-CB is still poorly understood. One of the known late-CB components is a testis-specific serine/threonine-protein kinase 2 (TSSK2), which localizes together with testis-specific serine/threonine-protein kinase 1 (TSSK1) to both the late-CB ring and satellite structures (Nayak *et al.* 1998, Shang *et al.* 2010) (Fig. 1A). TSSK1 and TSSK2 are expressed only in the testis in humans (Hao *et al.* 2004) and in mice (Bielke *et al.* 1994, Nayak *et al.* 1998), and function as serine kinases capable of phosphorylating testis-specific kinase substrate (TSKS) (Kueng *et al.* 1997). TSSK2 expression is specific to elongating spermatids, while TSSK1 can also be detected in spermatogonia and spermatocytes (Nayak *et al.* 1998, Li *et al.* 2011) in mice. Shang *et al.* created a double-knockout mouse model for *Tssk1* and *Tssk2* (*Tssk1/2* knockout), and showed that these genes are required for normal spermatid elongation and male fertility (Shang *et al.* 2010). Depletion of the *Tssk1/2* caused the absence of the late-CB ring and satellite and failure in mitochondrial loading to the elongating sperm tail (Shang *et al.* 2010). Knockout of TSKS caused similar defects in mitochondrial sheath formation and failure in eliminating the excess cytoplasm in spermiation (Shimada *et al.* 2023). These results suggested a functional relationship between the late-CB and the formation of sperm tail accessory structures.

While TSSK1 and TSSK2 are known to be vital for late-CB formation and correct sperm development, it is still unclear whether the late-CB retains the function of the CB in RNA regulation. Our research aimed to further explore the function of the late-CB by clarifying the late-CB composition and its association with RNA regulatory processes using TSSK2 as a late-CB marker.

## Materials and methods

### Mice

Adult mice from the C57BL/6 strain were used in all experiments. Mice were housed at the Central Animal Laboratory of the University of Turku, and the Finnish Animal Ethics Committee approved all experiments.

### Immunofluorescence and imaging

Adult (>8-week-old) mice testes were dissected and fixed in 4% paraformaldehyde (PFA) for 20 h at room temperature (RT), dehydrated, processed, and embedded in paraffin. 5 μm thick sections were prepared with a microtome and placed onto SuperFrost object slides. Sections were dried at 37°C o/n and stored at +4°C before staining. Testis sections were deparaffinized and rehydrated in a decreasing ethanol series before heat-induced antigen retrieval in Tris–EDTA buffer, pH 9.0, for 20 min. Unspecific sites were blocked using CAS-Block (Invitrogen, UK; cat code 00-8120) at RT for 1 h. Primary antibodies for TSSK2 (mouse, Abnova, cat code H00023617-M01, 0.5 μg/mL), SPATA19 (rabbit, ProteinTech, UK; cat code 16656-1-AP, 1 μg/mL), MIWI (rabbit, Cell Signaling Technology, The Netherlands; cat code 2079S, 1:500), EIF3A (rabbit, Nordic BioSite, Sweden; cat code BT-E1F0D7, 5 μg/mL), RPL24 (rabbit, ProteinTech, cat code 17082-1-AP, 0.9 μg/mL), and EIF4G3 (rabbit, Bethyl Laboratories, Germany; cat code A301-769A, 2 μg/mL) were diluted in Antibody Diluent for IHC (AbD, BD Pharmingen, USA; cat code 559148), and 50 μL of antibody dilution was incubated on testis sections at +4°C o/n. Negative control sections were incubated without a primary antibody. Sections were washed three times with PBS with 0.1% Tween 20 (PBST) for 5 min each, and secondary antibodies anti-mouse/rabbit IgG (donkey, Thermo Fisher Scientific, cat codes A21202 and A31573, 4 μg/mL) were diluted in AbD, added onto sections, and incubated at RT for 1 h. After washes, nuclei were stained using 4′,6-diamidino-2-phenylindole (DAPI), and slides were mounted with Vectashield with DAPI (Vector Laboratories, USA; cat code H-1200-10). Slides were imaged using the 3i CSU-W1 Spinning Disk (Intelligent Imaging Innovations) using SlideBook6 digital microscopy software and a Photometric Prime BSI sCMOS (2,048 × 2,048 pixels) camera. The objective used was a 63×/1.4 Oil Zeiss Plan-Apochromat. Alexa Fluor 488 was imaged with a 488 nm, 150 mW; Alexa Fluor 647 with a 640 nm, 100 mW; and DAPI with a 405 nm, 100 mW solid-state lasers. Filters used were 525/30, 692/40, and 445/45 nm, respectively. The pinhole was set to 50 μm. Images were acquired by taking a z-stack of 19 slices with 270 nm spacing, and figures were prepared using Fiji (Schindelin *et al.* 2012). Figures are presented as maximum projections, and the brightness and contrast were adjusted in all figures.

### Seminiferous tubule culture and translation assay

Stage IX-XI and XII-II seminiferous tubules were dissected from decapsulated adult mouse testes and placed into a flat-bottom 96-well plate well in 200 μL of Dulbecco’s Modified Eagle Medium (DMEM, Gibco, USA; cat code 21013024) supplemented with 500 μM l-azidohomoalanine (Click-iT™ AHA Alexa Fluor™ 488 Protein Synthesis HCS Assay, AHA, Thermo Fisher Scientific, cat code C10289). 300 μM cycloheximide (CHX, Merck Life Sciences, Germany; cat code C4859-1ml) was added to the control wells. Tubules were incubated at +34°C, 5% CO_2_ for 20 min and transferred onto an object slide for squashing (Kotaja *et al.* 2004). Slides were fixed in ice-cold acetone for 5 min and washed in PBS containing 3% bovine serum albumin (BSA) two times for 10 min. Cells were permeabilized in 0.2% Triton X-100 in PBS at RT for 15 min, and the newly synthesized peptides were labeled with Click-iT detection reagent according to the manufacturer’s protocol. After Click-iT labeling, slides were washed and stained with TSSK2 antibody at +4°C o/n as described above.

### Co-immunoprecipitation and western blotting

Testes were collected in 1 mL of cold lysis buffer (1% Triton X-100 in PBS, pH 7.4) supplemented with inhibitors including 0.2 mM phenylmethylsulfonyl fluoride, 1 mM dithiothreitol, and cOmplete Mini, EDTA-free protease inhibitor cocktail (Roche, Switzerland; cat code 11836170001), and homogenized with a Dounce homogenizer on ice. Lysates were incubated on ice for 30 min and centrifuged at 16,000 ***g*** at +4°C for 20 min. Magnetic Protein G Dynabeads (Thermo Fisher Scientific, cat code 10003D) were used to perform co-immunoprecipitation (co-IP) and pre-clearing of the lysate. Lysates were incubated with pre-washed beads on a vertical rotator at +4°C for 30 min. A 20 μL sample of the precleared lysate was saved for western blotting. 5 μg of TSSK2, DDX4 (Abcam, UK; cat code ab13840), and negative control antibody (mouse IgG: Invitrogen, cat code 31903; rabbit IgG: Invitrogen, cat code 10500C) were added to the Dynabeads and incubated at RT for 2 h. Dynabeads-antibody complexes were washed twice with PBST, and the precleared lysate was divided equally between the TSSK2 and IgG antibody-coupled beads and incubated on a vertical rotator at +4°C o/n. After incubation, a 20 μL sample of each co-IP supernatant sample (LO) was collected for western blotting, and Dynabeads were washed three times for 5 min and finally resuspended in 30 μL of lysis buffer. For SDS-PAGE electrophoresis, 4× SDS sample buffer was added to all samples and incubated at +95°C for 5 min before loading onto 4–20% Mini-PROTEAN TGX precast gels (Bio-Rad, USA; cat code 4561093). Proteins were size-separated at 15 mA constant current before the transfer to polyvinylidene difluoride membranes using the Trans-Blot Turbo Transfer Pack (Bio-Rad, cat code 1704156) and the Trans-Blot Turbo Transfer System (Bio-Rad) with the STANDARD SD protocol. After the protein transfer, the membranes were blocked with EveryBlot Blocking Buffer (Bio-Rad, cat code 12010020) for 10 min, followed by incubation with primary antibodies for EIF3A (5 μg/mL), TSSK2 (0.5 μg/mL), DDX4 (1 μg/mL), or RPL24 (0.45 μg/mL) in EveryBlot Blocking Buffer at +4°C o/n. Membranes were washed five times for 5 min with Tris-buffered saline with 0.1% Tween-20 (0.1% TBST) and then incubated in a secondary antibody solution with 1:5,000 horseradish peroxidase-conjugated anti-rabbit/mouse IgG (Cell Signaling Technologies, cat codes 7074 and 7076) in EveryBlot Blocking Buffer for 1 h at RT. After washes, membranes were developed for 1 min using Western Lightning ECL Pro Kit (PerkinElmer, USA; cat code NEL120E001EA) and imaged with Sapphire Biomolecular Imager (Azure Biosystems, USA).

### Mass spectrometry

For mass spectrometric (MS) analysis, Dynabead-antibody-protein complexes (*n* = 2, prepared as described above) were washed three times with 50 mM Tris buffer, pH 8, at +4°C and stored at −20°C before sample preparation and analysis at the Turku Proteomics Facility. The LC-ESI-MS/MS analyses were performed on a nanoflow HPLC system (Easy-nLC1200, Thermo Fisher Scientific) coupled to the Q Exactive HF mass spectrometer (Thermo Fisher Scientific, Germany) equipped with a nano-electrospray ionization source. Peptides were first loaded on a trapping column and subsequently separated inline on a 15 cm C18 column (75 μm × 15 cm, ReproSil-Pur 5 μm 200 Å C18-AQ, Dr Maisch HPLC GmbH, Germany). The mobile phase consisted of water with 0.1% formic acid (solvent A) and acetonitrile/water (80:20 (v/v)) with 0.1% formic acid (solvent B). Peptides were eluted with a linear 40 min gradient from 8 to 43% of eluent B. MS data were acquired automatically by using Thermo Xcalibur 4.1 software (Thermo Fisher Scientific). An information-dependent acquisition method consisted of an Orbitrap MS survey scan of the mass range 350–1,750 *m*/*z*, followed by HCD fragmentation of the ten most intense peptide ions. Data files were searched for protein identification using Proteome Discoverer 2.4 software (Thermo Fisher Scientific) connected to an in-house server running Mascot 2.7.0 software (Matrix Science). Data were searched against a SwissProt (version 2020) database using a mouse taxonomy filter. The Fixed Value PSM Validator node was used to assign confidence to PSMs based on fixed score thresholds. TSSK2-specific interaction candidate proteins, which were present only in TSSK2 specific co-IP samples, were selected. Hits with less than two peptides per identified candidate were removed from the analysis.

### Polysome fractionation

A 10–50% continuous sucrose gradient was prepared by carefully overlaying 10, 20, 30, 40, and 50% sucrose solutions on top of each other, followed by diffusion overnight at +4°C. Sucrose solutions were prepared in a lysis buffer containing 150 mM potassium acetate, 5 mM magnesium acetate, 2 mM DTT, cOmplete EDTA-free Protease Inhibitor Cocktail (Roche, 11873580001), 80 U/mL RiboLock RNase Inhibitor (Thermo Scientific, EO0382), and 50 mM HEPES, pH 7.5. Mouse testes were lysed in the lysis buffer supplemented with 100 μg/mL cycloheximide (Sigma-Aldrich, USA; C4859), 0.5% Triton X-100, and 0.25 M sucrose, with two 5 mm stainless steel beads (Qiagen, Germany; 69990), using TissueLyser LT (Qiagen) at 50 oscillations per second for 1 min, followed by 30 min incubation on ice. Testis lysates were then centrifuged at 500 *g* for 15 min at +4°C to remove tissue debris. Lysates were loaded on top of the 10–50% sucrose gradient and centrifuged at 153,300 ***g*** for 3 h at +4°C using an SW 32.1 Ti rotor (Beckman Coulter, 369651) and 17 mL Open-Top Thinwall Ultra-Clear Tubes (Beckman Coulter, C14297). 1 mL fractions of the gradients were collected. Absorbances at 260 nm were measured using a NanoDrop ND-1000 Spectrophotometer (Thermo Scientific) to determine free RNP, monosome, and polysome fractions. 50 μL of a 1 mL fraction were collected for western blotting, supplemented with SDS sample buffer (4% SDS, 20% glycerol, 10% 2-mercaptoethanol, 0.004% bromophenol blue, and 0.125 M Tris HCl), and stored at −20°C. For practical reasons, samples for western blotting were collected only from every other fraction (1, 3, and 5).

### Cross-linking and immunoprecipitation of RNA-protein complexes

Adult (>8-week-old) mice testes were dissected, decapsulated, and seminiferous tubules were minced with scissors before digestion with 0.05% collagenase type I (Worthington, cat code LS004196) in 0.1% sucrose-PBS solution in an end-over-end rotator at RT for 1 h. To remove bigger parts of the seminiferous tubules, the germ cell suspension was filtered through a 100 μm cell strainer, pelleted by centrifugation at 600 ***g*** for 5 min, and washed once with cold PBS. Germ cells were cross-linked in 10 mL of 0.2% PFA-PBS in an end-over-end rotator at RT for 20 min, and cross-linking was stopped by adding 1 mL of 2.5 M glycine and rotating at RT for 5 min. Germ cells were pelleted by centrifugation at 600 ***g*** for 5 min, washed once with cold PBS, and lysed in lysis buffer (50 mM Tris–HCl, pH 7.5, 1% Triton X-100, 0.5% sodium deoxycholate, 0.05% sodium dodecyl sulfate, 1 mM ethylenediaminetetraacetic acid, 150 mM NaCl, 1 × cOmplete protein inhibitor cocktail, 0.2 mM phenylmethylsulfonyl fluoride, 1 mM dithiothreitol, and 0.04 U/mL RiboLock RNase Inhibitor (Thermo Fisher Scientific, cat code EO0381)). The lysate was sonicated with a Bioruptor UCD-200 sonicator (Diagenode) at medium settings for 6 × 30 s with 30 s intervals, and the lysate was cleared by centrifugation at 1,000 ***g*** for 10 min at +4°C. The supernatant was discarded, and the pellet was suspended in lysis buffer and sonicated with the Bioruptor at medium settings for 2 × 30 s with 30 s intervals. Pellet fractions were used for co-IP as described above to pull down TSSK2- and DDX4-specific protein-RNA complexes.

### Electron microscopy

For ultrastructural analysis, the above-described Dynabeads-protein-RNA complexes were fixed with 5% glutaraldehyde, treated with potassium ferrocyanide-osmium fixative, embedded in epoxy resin, and stained with 1% uranyl acetate and 0.3% lead citrate. Ultrathin sections of Dynabeads–protein complexes were imaged with a JEM-1400 Plus transmission electron microscope and an OSIS Quemesa 11 mPix bottom-mounted digital camera. Fiji was used for image preparation.

### RNA extraction from the protein-RNA-complexes

The Dynabeads-protein-RNA complexes described above were diluted in 30 μL of lysis buffer and reverse cross-linked at +70°C for 45 min. Reverse cross-linked samples were mixed with 800 μL of TRIsure (Bioline, UK; cat code BIO-38033), incubated for 5 min at RT, followed by RNA phase separation from DNA and proteins with chloroform and precipitation with isopropanol. Purified RNA pellets were washed twice with ice-cold 75% ethanol and dissolved in nuclease-free water. RNA was analyzed using the Bioanalyzer RNA 6000 Pico assay kit and the 2100 Bioanalyzer Instrument (Agilent), and samples with >40 ng of total RNA were qualified for library preparation (*n* = 2).

### RNA sequencing and data analysis

Directional libraries without rRNA removal were prepared for two anti-TSSK2 IPs and two anti-DDX4 IPs and sequenced on the Illumina NovaSeq 6000 platform using a paired-end 150 strategy at Novogene (UK) Company Limited. The quality of raw reads was evaluated by FastQC (v0.11.9). Adapters were removed from sequences, and low-quality bases were cut using Trimmomatic (v0.39) (Bolger *et al.* 2014). STAR (v2.7.1a) was used to map the clean reads to the mouse reference genome (Ensembl: Mus musculus.GRCm38.101) (Dobin *et al.* 2013). The reads were then assigned and counted using featureCounts (v2.0.3) (Liao *et al.* 2014). Raw reads were normalized and analyzed using DESeq2 (v1.40.1) (Love *et al.* 2014). The GO enrichment analysis was performed using the topGO package (v2.52.0). The transcriptome datasets of different germ cell types during mouse spermatogenesis (GSE35005) and polysome fractions of pachytene spermatocytes and elongating spermatids (GSE80353) were downloaded from the NCBI GEO database (Gan *et al.* 2013, Zhang *et al.* 2017). Mapping of reads and normalization for these two datasets were performed similarly to the TSSK2 IP samples. Gene functional classification was done using DAVID (Sherman *et al.* 2022).

### *In situ* hybridization

Adult mouse testes were fixed in 4% paraformaldehyde (PFA) o/n, followed by dehydration and embedding in paraffin. Sections were cut at 5 μm thickness and mounted onto SuperFrost Plus slides. mRNA detection was performed using the RNAscope® Multiplex Fluorescent Reagent Kit v2 (Advanced Cell Diagnostics, ACD, USA) following the manufacturer’s instructions.

Briefly, sections were deparaffinized, treated with hydrogen peroxide, and antigen retrieval was performed using target retrieval buffer at 100°C for 15 min. Protease digestion was carried out at 40°C for 30 min. Slides were then hybridized with RNAscope probes targeting *Dcdc2c* and *Tmem249* mRNAs. Signal amplification and fluorophore development were performed according to the kit instructions. Finally, nuclei were stained with DAPI and mounted using ProLong Gold Antifade Mountant (Thermo Fisher). Imaging was performed using a confocal fluorescence microscope (3i Marianas CSU-W1 spinning disk) and analyzed using ImageJ software.

## Results

### Late-CB dynamics during spermiogenesis

We first characterized the localization of TSSK2 during spermatogenesis in detail by performing immunofluorescence analysis on mouse testis sections. TSSK2 localized to the cytoplasmic granules in elongating spermatids corresponding to the late-CB, and the staining pattern was comparable to previously published results (Shang *et al.* 2010). Subsequently, the TSSK2-positive late-CB split into two separate granules, the ring and the cytoplasmic satellite, in step 10 spermatids (Fig. 1B). The ring stayed in close contact with the neck until steps 13–14 and then traveled along the sperm tail simultaneously with mitochondrial loading to the midpiece (Fig. 1B). Just before spermiation in step 16 spermatids, the ring disappeared, but the TSSK2 signal could still be detected in the acrosomal area (Fig. 1B). The satellite was clearly visible in the cytoplasm of step 11–16 spermatids (Fig. 1B).

### TSSK2 forms complexes with proteins involved in RNA regulation and translation

**Figure 2:**
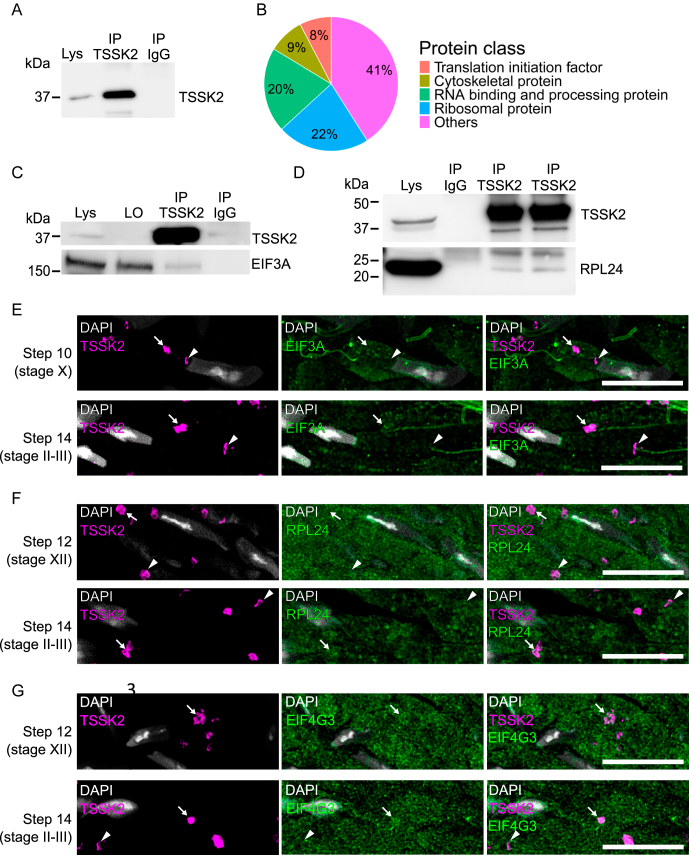
TSSK2 associates with translation factors. (A) Western blotting to validate successful immunoprecipitation of TSSK2 (IP TSSK2) for mass spectrometry. The image shows one representative sample submitted to mass spectrometry. Mouse IgG was used as a negative control for the IP (IP IgG). Lys: input lysate. (B) Mass spectrometric identification of TSSK2-interacting proteins in the testis. Pie chart shows the most common protein classes among TSSK2-interacting proteins according to the Panther Classification System. See also Supplementary Table S1. (C) Validation of the interaction between TSSK2 (38 kDa) and EIF3A (167 kDa) by western blotting after immunoprecipitation of TSSK2 from the testis. Lys: input lysate, LO: leftover lysate after IP, IP TSSK2: anti-TSSK2 IP, IP IgG: negative control IgG IP. (D) Validation of the interaction between TSSK2 and RPL24 (18 kDa) by western blotting. (E) Immunofluorescence staining of PFA-fixed paraffin-embedded testis section using TSSK2 (magenta) and EIF3A (green) antibodies. Step 10 (stage X) and step 14 (stage II) elongating spermatids are shown. DAPI stains the nuclei (gray). EIF3A signal often accumulates in the late-CB satellite (arrow). In step 14 spermatids, the late-CB ring surrounds the EIF3A-positive principal piece of the sperm tail (arrowhead). (F) Immunofluorescence staining using TSSK2 (magenta) and RPL24 (green) antibodies. Step 12 (stage XII) and step 14 (stage II) elongating spermatids are shown. (G) Immunofluorescence staining using TSSK2 (magenta) and EIF4G3 (green) antibodies. Step 12 (stage XII) and step 14 (stage II) elongating spermatids are shown. (E–G) White arrowheads: TSSK2-positive late-CB rings, white arrows: TSSK2-positive late-CB satellites. Scale bar: 10 μm. See Figs S1 and S2 for broader views.

To gain insight into the function of the TSSK2-positive late-CB, we immunoprecipitated TSSK2 complexes from the mouse testis to identify TSSK2-interacting proteins by mass spectrometry (Fig. 2A). We identified 288 proteins with more than two peptide hits in both replicates and no hits in the control IgG immunoprecipitation (IP) (Supplementary Table S1 (see section on Supplementary materials given at the end of the article)). TSSK2 was detected in both IP samples together with its known interaction partners TSSK1 and TSKS (Xu *et al.* 2008, Shang *et al.* 2010), validating the success of the IP. Furthermore, comparison of TSSK2-interacting proteins with the previously identified TSKS-interacting proteins (Shimada *et al.* 2023) revealed that ten out of fourteen identified TSKS-interacting proteins (TSKS, TSSK1B, HSPA8, TSSK2, ODF1, CCDC91, UHRF1BP1L, HSPAL, HSPA5, and KRT5) were also found in the TSSK2 complexes. The classification of the TSSK2-interacting proteins with the Panther Classification System revealed that a majority of them were proteins involved in RNA regulation, RNA metabolism, and translation (Fig. 2B). Fifty-nine (20%) of the TSSK2-interacting proteins were classified as RNA-binding proteins and proteins involved in RNA processing (‘RNA metabolism proteins’, ‘RNA helicases’, ‘mRNA capping factors’, ‘RNA splicing factors’, ‘mRNA polyadenylation factors’). Twenty-two proteins (8%) were classified as ‘translation initiation factors’, and 64 (22%) as ribosomal proteins (Supplementary Table S1). Twenty-seven proteins (9%) were shared with the CB proteome (Meikar *et al.* 2014). These findings clearly link the function of TSSK2 and the late-CB to RNA regulation and translational activity in elongating spermatids.

### Translation-related proteins associate with TSSK2-positive late-CBs in elongating spermatids

Interestingly, we found 11 out of the 13 subunits of the eukaryotic translation initiation factor 3 (EIF3, EIF3A, -B, -C, -D, -E, -F, -G, -H, -I, -K and -L, Supplementary Table S1) as TSSK2-interacting proteins. The EIF3 complex is a multiprotein complex regulating mRNA translation from initiation to elongation (Wolf *et al.* 2020). The EIF3A subunit is the largest subunit in the EIF3 complex with a known role in recruiting mRNAs to the pre-initiation complex (Yin *et al.* 2018, Stanciu *et al.* 2022); therefore, it was selected for further studies. The interaction of TSSK2 with EIF3A was validated by immunoprecipitation followed by western blotting (Fig. 2C). We also validated the interaction of TSSK2 with one of the ribosomal subunits, RPL24, which was identified as a TSSK2-interacting protein in the mass spectrometry analysis (Fig. 2D).

Next, we performed co-immunofluorescence analysis of TSSK2 with selected translation regulators to understand the relationship between the late-CB and translation. In addition to EIF3A and RPL24, the translation elongation factor EIF4G was selected for the analysis. All three translation factors were found to be expressed during all steps of spermatogenesis, with relatively strong expression in elongating spermatids, indicating high translational activity in these cells (Supplementary Figs S1 and S2). These proteins were found to be widely distributed in the cytoplasm. However, some signal was also found to be accumulated in the TSSK2-positive granules, or in their vicinity, particularly in the late-CB satellite (Fig. 2E, F, G). Interestingly, EIF3A signal was also found in the sperm tail, which is surrounded by the TSSK2-positive late-CB ring (Fig. 2E). These results provide proof that translation-related proteins are co-expressed with TSSK2 in elongating spermatids, where they make frequent contacts with the TSSK2-positive late-CB.

### TSSK2-positive late-CB associates with translational activity

**Figure 3:**
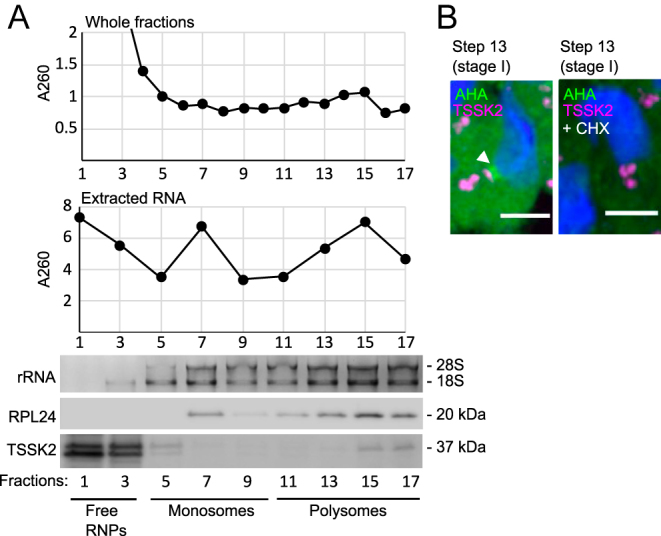
TSSK2-positive late-CBs associate with translational activity in elongating spermatids. (A) Polysome fractionation from total mouse testes. The graphs show the absorbance at A260 of the collected fractions (upper graph) or RNA extracted from the fractions (lower graph). Distribution of ribosomes within the fractions was visualized on agarose gels (rRNA). The presence of RPL24 and TSSK2 was visualized by western blotting. (B) Cultured seminiferous tubules were incubated with modified methionine L-azidohomoalanine (AHA), and the localization of the newly synthesized peptides was analyzed by Click-iT AHA Alexa Fluor 488 assay. Newly synthesized peptides (green, arrowhead) accumulated between the TSSK2-positive late-CB ring (magenta) and the basal body in step 13 spermatids. Cycloheximide (CHX), which blocks protein translation and AHA incorporation into peptides, was used as a negative control. DAPI (blue) stains the nuclei. Scale bar 10 μm.

Next, we wanted to explore whether, in addition to forming complexes with translation-related proteins, TSSK2 associates with translational activity. To this end, we performed polysome fractionation from adult mouse testes and showed that, although the majority of TSSK2 was found in the free RNP fraction, TSSK2 was also detected in the polysome fraction together with RPL24, supporting the involvement of TSSK2 in translation (Fig. 3A). To further monitor the association of translational activity with the TSSK2 granules, we set up the seminiferous tubule culture assay and labeled nascent peptides with modified methionine detectable by Click chemistry (Fig. 3B). As a negative control, we used tubules incubated with cycloheximide (CHX) to inhibit translation. Translational activity was shown to be active in elongating spermatids, as demonstrated by the strong cytoplasmic labeling of newly synthesized proteins. Interestingly, in step 13 spermatids, the signal accumulated in a focus next to the TSSK2-positive late-CB ring (Fig. 3B). While it remains unclear whether translation takes place in the late-CB or its vicinity, our findings support the role of the late-CB function in translational regulation in elongating spermatids.

### TSSK2 binds a specific set of mRNAs

**Figure 4:**
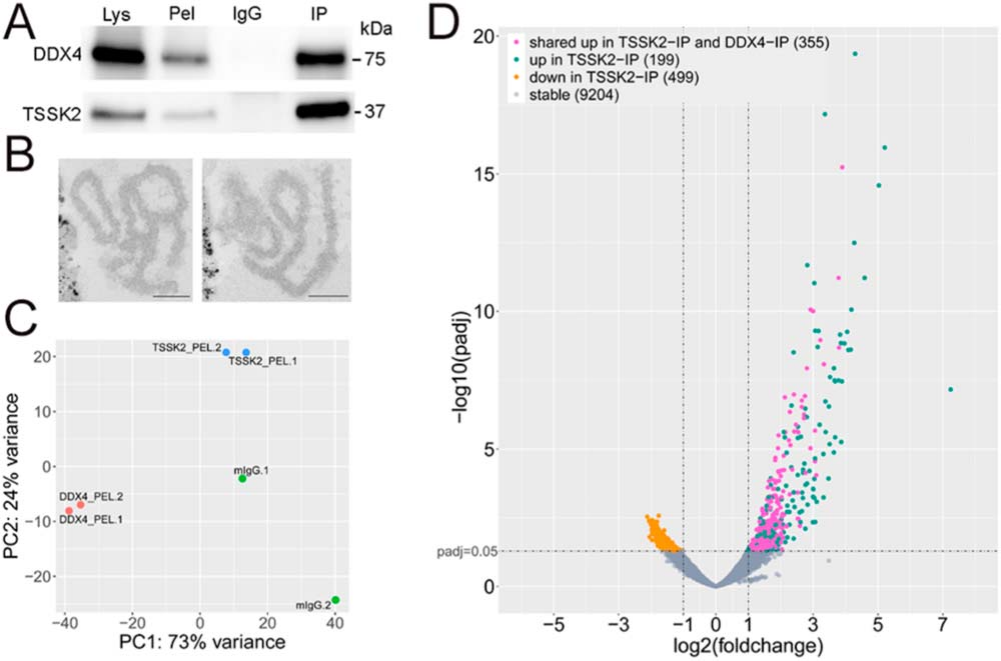
TSSK2 associates with a specific pool of mRNAs in elongating spermatids. (A) Western blotting to validate successful anti-DDX4 and anti-TSSK2 IP from the mouse cross-linked testicular lysate. Lys: lysate after cross-linking and sonication, Pel: pellet fraction after low-speed centrifugation (used as input for the IP), IgG: negative control IP, IP: IP with specific antibodies (upper panel: anti-DDX4, lower panel: anti-TSSK2). (B) Electron microscopy of the Dynabead-protein complex after anti-TSSK2 IP showing the electron-dense late-CB granule. Two representative example images are shown. Scale bar 200 nm. (C) PCA plot shows RNAseq sample clustering. (D) Volcano plot showing the results of the differential expression analysis to compare mRNA levels in TSSK2-IP vs control IgG-IP. Upregulated mRNAs (Log_2_FC ≥ 1, P-adj ≤ 0.05) are considered TSSK2-associated mRNAs (*n* = 554). mRNAs that are also found upregulated in DDX4-IP vs IgG-IP (Log_2_FC ≥ 1, P-adj ≤ 0.05) are indicated with different color (magenta, *n* = 355). See also Supplementary Table S2.

The interaction of TSSK2 with proteins involved in RNA metabolism and translation prompted us to study whether the late-CB contains mRNAs that could possibly be released for temporally regulated translation in elongating spermatids. To this end, we immunoprecipitated TSSK2 complexes from cross-linked testicular lysate to identify TSSK2-associated mRNAs by RNA sequencing (RNAseq). We chose the cross-linking approach because of our earlier experience with CB isolation from round spermatids, which requires cross-linking to keep the CB structure intact (Meikar & Kotaja 2014). In the CB isolation, the CBs are immunoprecipitated with an anti-DDX4 antibody from the pellet fraction after low-speed centrifugation of the cross-linked lysate. For the late-CB isolation, we followed the same approach using an anti-TSSK2 antibody. Western blotting validated the successful immunoprecipitation (Fig. 4A). Furthermore, we confirmed the purification of intact late-CBs by electron microscopy, which visualized the lobular, electron-dense structure resembling the late-CB attached to the Dynabeads (Fig. 4B). These structures present either the late-CB satellite or the ring, which cannot be differentiated based on this analysis.

To identify TSSK2-associated mRNAs, we sequenced two TSSK2-IP samples and two control IgG-IP samples. In addition, we sequenced two DDX4-IP samples (the CB samples) as controls. The PCA plot demonstrated the separation of the samples into three experimental groups (Fig. 4C). The comparison of the TSSK2-IP to the control IgG-IP revealed 554 mRNAs that were found at least two-fold more in TSSK2-IP (log_2_FC ≥ 1, P-adj ≤ 0.05) (Fig. 4D, Supplementary Table S2). We also identified DDX4-associated mRNAs by comparing DDX4-IP to the control IgG-IP and showed that 355 out of 554 TSSK2-interacting mRNAs were also found in DDX4-positive CBs (Fig. 4D, Supplementary Table S2). These results further validated the function of TSSK2 and the late-CB in RNA regulation and suggest a functional relationship between the CB and the late-CB.

### TSSK2-associated mRNAs encode proteins needed for late spermiogenesis

**Figure 5:**
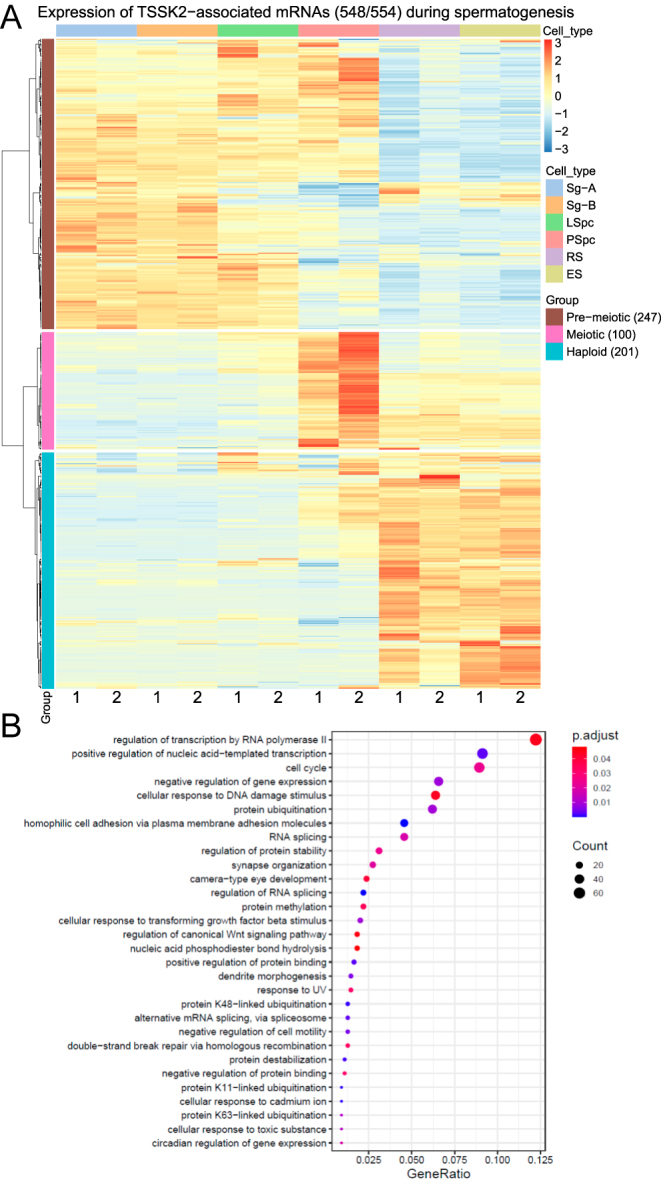
Expression profile and GO enrichment analysis of TSSK2-associated mRNAs. (A) Heat-map shows the expression of TSSK2-associated mRNAs (*n* = 548) during spermatogenesis in type A spermatogonia (Sg-A), type B spermatogonia (Sg-B), leptotene spermatocytes (LSpc), pachytene spermatocytes (PSpc), round spermatids (RS), and elongating spermatids (ES) (GSE35005). mRNAs were clustered into three groups according to their peak expression. Pre-meiotic group: expression peaks in premeiotic and early meiotic cells (247 mRNAs), Haploid group: expression peaks in spermatids (201 mRNAs), Meiotic group: expression peaks in pachytene spermatocytes (100 mRNAs). The Ward.D2 clustering method was used. See also Supplementary Table S3 and Fig. S3. (B) GO enrichment analysis of the TSSK2-associated mRNAs. The first 30 Biological Processes were selected for visualization based on GeneRatio (the number of genes associated with the GO term divided by the total number of genes). p.adjust: adjusted *P*-values indicated by a gradient scale, with red indicating the most significant values. Count: the number of genes associated with the GO term is illustrated by a dot with a proportional size according to the number of associated genes found in our gene list. See also Supplementary Tables S4, S5, S6, S7.

To characterize the TSSK2-associated mRNAs, we first studied their expression during spermatogenesis using a published dataset containing RNAseq data from different spermatogenic cell types. Six mRNAs were discarded from the analysis because they were not found to be expressed in spermatids. We showed that the 548 TSSK2-associated mRNAs had distinct expression patterns, with expression peaking at specific phases of spermatogenesis. The expression of 201 mRNAs (‘Haploid’; 201/548, 37%) peaked in round spermatids and elongating spermatids, coinciding with the presence of the late-CB (Fig. 5A, Supplementary Table S3). We selected two of these mRNAs, *Tmem249* and *Dcdc2c*, to validate their expression pattern by *in situ* hybridization (Supplementary Fig. S3). The expression of these selected mRNAs was shown to peak in late round spermatids (*Tmem249*) or early elongating spermatids (*Dcdc2c*), and the downregulation of their expression coincided with the appearance of the late-CB (Fig. 1 and Supplementary Fig. S3). Other TSSK2-associated mRNAs were expressed in round spermatids and elongating spermatids in RNAseq data, but their expression peaked in pre-meiotic and early meiotic cells (‘Pre-meiotic’; 247/548, 45%) or in pachytene spermatocytes (‘Meiotic’; 100/548, 18%) (Fig. 5A).

GO enrichment analysis of the TSSK2-associated mRNAs revealed enrichment of mRNAs whose function is connected to the regulation of transcription and RNA splicing, cell cycle, DNA damage, ubiquitination, and cell adhesion (Fig. 5B, Supplementary Table S4). To get insight into the functional differences between TSSK2-associated mRNAs with either pre-meiotic, meiotic, or haploid expression peaks (Fig. 5A), we performed the GO enrichment analysis separately for each group (Supplementary Figs S4, S5, S6, Supplementary Tables S5, S6, S7). Interestingly, GO terms related to cell cycle, regulation of transcription, splicing, and DNA damage were specifically enriched among mRNAs with ‘Pre-meiotic’ expression peak (247), while cell adhesion and ubiquitination were overrepresented among mRNAs with ‘Haploid’ expression peak (201).

**Figure 6:**
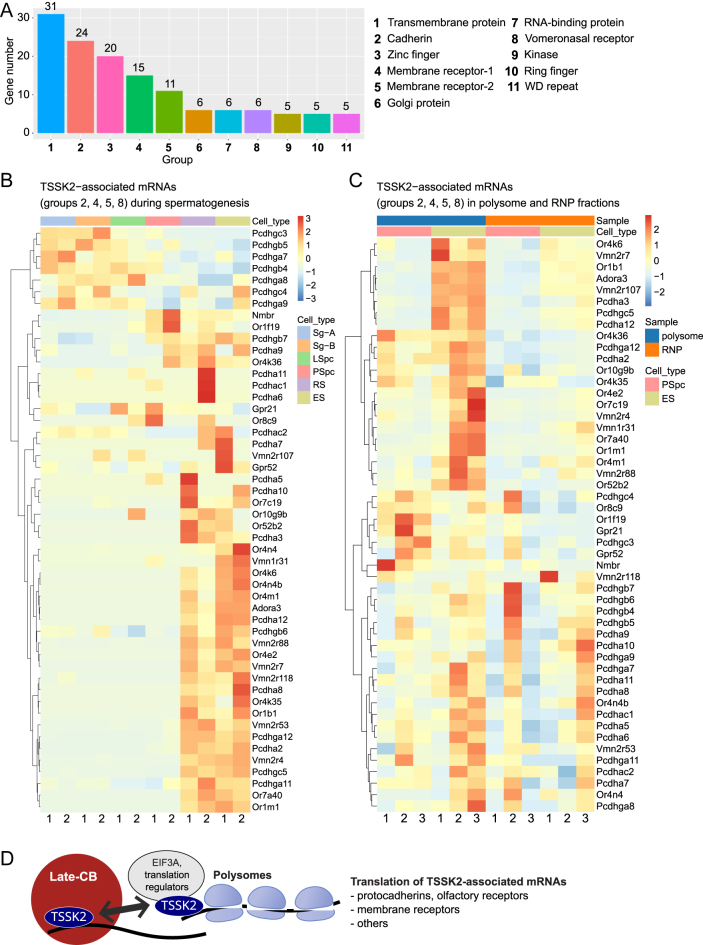
Late-translating membrane proteins are highly represented among TSSK2-associated mRNAs. (A) Gene functional classification of the TSSK2-associated mRNAs using the highest stringency setting in the DAVID functional classification tool. The analysis identified 11 functional groups, including 128 out of 548 analyzed mRNAs. Groups were named according to the common annotations of mRNAs within the group. The bar plot shows the number of mRNAs clustered in each group. Six mRNAs were classified into two distinct groups. See also Supplementary Table S8. (B) Heat-map visualizes the expression patterns of mRNAs from four groups (‘Cadherin’, ‘Vomeronasal receptor’, ‘Membrane receptor 1’, ‘Membrane receptor 2’) during spermatogenesis. Sg-A, type A spermatogonia; Sg-B, type B spermatogonia; LSpc, leptotene spermatocytes; PSpc, pachytene spermatocytes; RS, round spermatids; ES, elongating spermatids. See also Supplementary Table S3. (C) Heat-map visualizes the abundance of mRNAs from the same groups as in (B) in polysome and RNP fractions isolated from pachytene spermatocytes (PSpc) and elongating spermatids (ES) (GSE80353). In both (B) and (C), the Ward.D2 clustering method was used. See also Supplementary Table S9. (D) Schematic drawing illustrates the main findings of the study. TSSK2 and TSSK2-associated mRNAs accumulate in the late-CB, but also in the polysome fraction, indicating the involvement of the late-CB in the regulation of translation of mRNAs required for late spermiogenesis, including cell membrane receptors and protocadherins.

Further functional classification of TSSK2-associated mRNAs using the DAVID tool (Sherman *et al.* 2022) identified 11 functional groups based on their common annotations (Fig. 6A, Supplementary Table S8). A closer examination of the functional groups revealed that TSSK2 associates with a high number of mRNAs encoding membrane-associated proteins. The largest group included 31 mRNAs encoding transmembrane proteins, and the second largest group included 24 protocadherin-encoding mRNAs. Two groups consisted of mRNAs encoding membrane receptors (11 and 15 mRNAs), with olfactory receptors being the predominant protein class within these groups (19 mRNAs). Interestingly, in addition to olfactory receptors, another type of sensory receptors, vomeronasal receptors, were highly represented among TSSK2-associated mRNAs (7 mRNAs).

Considering the potentially important role of cell surface receptors and adhesion molecules in sperm function, we selected the mRNAs from ‘Cadherin’, ‘Membrane receptor-1’, ‘Membrane receptor-2’, and ‘Vomeronasal receptor’ groups for further analysis. Using the same expression data as in Fig. 5A, we showed that the expression of TSSK2-associated mRNAs from these groups mainly peaked in late-CB-containing spermatids (Fig. 6B, Supplementary Table S3). We also used published RNAseq data on RNP and polysome fractions isolated from pachytene spermatocytes and elongating spermatids (GSE80353) (Zhang *et al.* 2017) to study if these mRNAs are actively translated in elongating spermatids. Interestingly, they were all found in the polysome fractions in elongating spermatids, and the majority of them were more abundant in the polysome fractions from elongating spermatids compared to polysome fractions from spermatocytes (Fig. 6C, Supplementary Table S9). Similarly to TSSK2 (Fig. 3C), TSSK2-associated mRNAs were also found in RNP fractions in addition to polysome fractions, but appeared to be more abundant in polysome fractions (Fig. 6C). These results support the involvement of TSSK2 and the late-CB in the temporally regulated translation of these late-translating mRNAs (Fig. 6D).

## Discussion

Regulated translation and uncoupling of transcription from translation are common during spermatogenesis, particularly during the haploid differentiation phase (Kleene 2003). The molecular content of the CB, which includes a wide range of mRNA-binding proteins as well as mRNAs that are known to be translated later in elongating spermatids, supports the role of the CB in the storage and translational repression of mRNAs (Lehtiniemi & Kotaja 2018). While the CB and late-CB share similar ultrastructure (Fawcett *et al.* 1970), their protein composition appears to be different, and the expression of the main core CB components, such as DDX4 and PIWIL1, is downregulated at the onset of the late-CB appearance (Da Ros *et al.* 2017, Lehtiniemi *et al.* 2022). Therefore, the functional relationship between the CB and the late-CB has remained unclear. Here we provide evidence for the first time that the function of the CB in RNA regulation is retained in the late-CB, as it binds mRNAs and translational regulators, including the EIF3 translation elongation complex.

Our results suggest that TSSK2 is associated with active translation. This was supported by the co-fractionation of TSSK2 with polyribosomes and the accumulation of newly synthesized polypeptide chains next to the TSSK2-positive late-CB in step 13 spermatids, preceding the midpiece formation and mitochondrial loading to the sperm tail. Localized translation is a powerful way to control proteomes with spatiotemporal precision in response to regional or microenvironmental cues (Buxbaum *et al.* 2015). Elongating spermatids undergo major cytodifferentiation, including the appearance and clearance of the microtubular structure, the manchette, the formation of outer dense fibers in the sperm tail, and the mitochondrial loading into the midpiece. These processes require coordinated action of a broad variety of specific proteins, highlighting the need for the regulation of their biosynthesis. Importantly, our results revealed the association of the late-CB component TSSK2 with the EIF3 complex and late-translating mRNAs. These findings link the function of the late-CB to the regulation of localized translation, possibly by releasing stored mRNAs for translation (Fig. 6D).

The localization of EIF3A to the tail of developing sperm indicates that the sperm flagellum could serve as a platform for localized protein synthesis to produce proteins required for the construction and function of sperm components. This has been shown to be the case in *Drosophila melanogaster*, where an RNP granule associated with the distal end of the elongating spermatid cyst provides axonemal dynein heavy chain mRNA for localized protein production required for tail formation (Fingerhut & Yamashita 2020). Localized translation is also evidenced in somatic cilia. Hao *et al.* revealed that cilia in multiciliary mouse ependymal cells contain mRNAs, several EIF3 complex members, as well as ribosomal proteins, and are actively synthesizing proteins to maintain cilia function (Hao *et al.* 2021). Moreover, they showed that FMRP, a known factor in mRNA transport, is required to deliver alpha-tubulin mRNA into cilia for translation (Hao *et al.* 2021).

In this study, we identified more than 500 mRNAs that were associated with TSSK2 in the mouse testis and are potentially regulated by late-CB-mediated mechanisms. A large part of them encodes membrane-associated proteins, whose translation usually occurs at the ribosomes on the membranes of the endoplasmic reticulum (ER). Along with other massive cellular transformations, the ER also undergoes major structural changes during spermatogenesis. Interestingly, while the ER is widely distributed in the cytoplasm in earlier cell types, it accumulates around the late-CB and becomes aligned along the flagellum in step 8–14 spermatids (Hermo *et al.* 2010), therefore enabling close communication with the late-CB. The CB in round spermatids has also been shown to have an intimate association with the cellular endomembrane system, including the ER (Soderstrom 1981, Da Ros *et al.* 2015). In addition to the ER membranes, proteins can also be translated on the surface of other organelles, such as mitochondrial outer membranes (Lashkevich & Dmitriev 2021). Considering the association of the late-CB with mitochondria during midpiece formation (Shang *et al.* 2010), it is also possible that the late-CB cooperates with mitochondria-localized translation.

Two classes of mRNAs, protocadherins and olfactory receptors, stood out in the analysis due to their abundance among the TSSK2-associated mRNAs (25 protocadherins and 18 olfactory receptors). Interestingly, only very few of these mRNAs (3 olfactory receptor mRNAs) were found in the CBs in round spermatids, indicating that their regulation is specific to the late-CB during late steps of spermiogenesis. Protocadherins are cell adhesion molecules, and all three protocadherin families (α, -β, and -γ) have been reported to be expressed in the rat testis (Johnson *et al.* 2000). The role of protocadherins in spermatogenesis and male fertility is still largely unexplored, but some family members have been shown to have specific localization patterns in elongating spermatids and mature sperm (Johnson *et al.* 2004, Anilkumar *et al.* 2017). Protocadherin expression was also correlated with fertility in men, highlighting their potential functional role in maintaining fertility (Anilkumar *et al.* 2017). Olfactory receptors have a better-established role in male reproduction, and they are known to localize to sperm and mediate sperm-egg chemotaxis (Ralt *et al.* 1991, Spehr *et al.* 2003). Our results showed that the expression of TSSK2-associated protocadherins and olfactory receptors peaked during late steps of spermatogenesis, where they were associated with actively translating polysomes, suggesting that TSSK2 and the late-CB are involved in their regulated translation to support the functional properties of sperm.

Altogether, our results elucidate the function of the late-CB in elongating spermatids, providing evidence that it takes part in the coordination of mRNA regulation and translation during late spermatogenesis. Keeping in mind that the disruption of the late-CB structure by the deletion of *Tssk1/Tssk2* genes in mice leads to severe flagellar defects (Shang *et al.* 2010), these late-CB-mediated functions are likely to play a critical role in the development of spermatozoa and, therefore, in the maintenance of male fertility.

## Supplementary materials





## Declaration of interest

N Kotaja is an Associate Editor of *Reproduction* and was not involved in the review or editorial process for this paper, on which she is listed as an author. The authors declare that there is no conflict of interest that could be perceived as prejudicing the impartiality of the work reported.

## Funding

This work was supported by the Jalmari and Rauha Ahokas Foundation (MSL), Research Council of Finlandhttps://doi.org/10.13039/501100002341 (NK: 315948, 361207; MSL: 321398), Jane and Aatos Erkko Foundation (NK), and Novo Nordisk Foundation (NK). AS was funded by BBSRC grant BB/V011251/1.

## Author contribution statement

MSL, AS, and NK conceived and designed the study. MSL, SK, SL, AA, OO, PT, and AS designed and performed laboratory experiments and analyzed data. LM and MB analyzed the RNA-seq data. MSL, LM, and NK interpreted the data and drafted the manuscript. All authors reviewed and edited the final version of the manuscript.

## Data availability

The sequencing data generated in this study have been deposited in the National Center for Biotechnology Information’s (NCBI) Gene Expression Omnibus (GEO) under the accession number GSE262643. The mass spectrometry proteomics data have been deposited to the ProteomeXchange Consortium via the PRIDE partner repository with the dataset identifier PXD065042.
