# Supplementary material for: The late chromatoid body component TSSK2 is involved in translational regulation in elongating spermatids in mice
Source: Reproduction. 2025 Oct 15;170(6):e250297. doi: 10.1530/REP-25-0297 (PMC12529751; doi:10.1530/REP-25-0297)
Supplement: Supplementary file 1 [file supplementary_figures.pdf]

The late chromatoid body component TSSK2 is involved in translational regulation in elongating spermatids in mice

Mari S. Lehti, Lin Ma, Salli Kärnä, Samuli Laasanen, Ammar Ahmedani, Opeyemi Olotu, Matthieu Bourgery, Panyi Tran, Anu Sironen, and Noora Kotaja

SUPPLEMENTARY INFORMATION

Supplementary Figures:

- **Supplementary Figures S1.** Colocalization of EIF3A and TSSK2 in adult mouse testes.
- **Supplementary Figure S2.** Colocalization of RPL24 and EIF4G3 with TSSK2 in adult mouse testes.
- **Supplementary Figure S3.** Expression of *Dcdc2c* and *Tmem249* mRNAs in adult mouse testes.
- **Supplementary Figure S4.** GO enrichment analysis of the 247 TSSK2-associated mRNAs in the "Pre-meiotic" expression group.
- **Supplementary Figure S5.** GO enrichment analysis of the 100 TSSK2-associated mRNAs in the "Meiotic" expression group.
- **Supplementary Figure S6.** GO enrichment analysis of the 201 TSSK2-associated mRNAs in the "Haploid" expression group.

Supplementary Tables:

- **Supplementary Table S1.** Mass spectrometric analysis of the TSSK2-interacting proteins in the mouse testis.
- **Supplementary Table S2.** Differential expression analysis of the mRNAs in TSSK2-IP vs. control IgG-IP.
- **Supplementary Table S3.** Expression of 548 TSSK2-associated mRNAs in different cell types during spermatogenesis.
- **Supplementary Table S4.** GO enrichment analysis of the 548 TSSK2-associated mRNAs.
- **Supplementary Table S5.** GO enrichment analysis of the 247 TSSK2-associated mRNAs in the "Pre-meiotic" expression group.
- **Supplementary Table S6.** GO enrichment analysis of the 100 TSSK2-associated mRNAs in the "Meiotic" expression group.
- **Supplementary Table S7.** GO enrichment analysis of the 201 TSSK2-associated mRNAs in the "Haploid" expression group.
- **Supplementary Table S8.** Gene functional classification of the 548 TSSK2-associated mRNAs.
- **Supplementary Table S9.** Abundance of 50 mRNAs from the functional classes “Cadherin”, “Vomeronasal receptor”, “Membrane receptor 1” and “Membrane receptor 2” (see Supplementary Table S8) in polysome and RNP fractions isolated from pachytene spermatocytes (PSpc) and elongating spermatids (ES).

Supplementary Figure S1

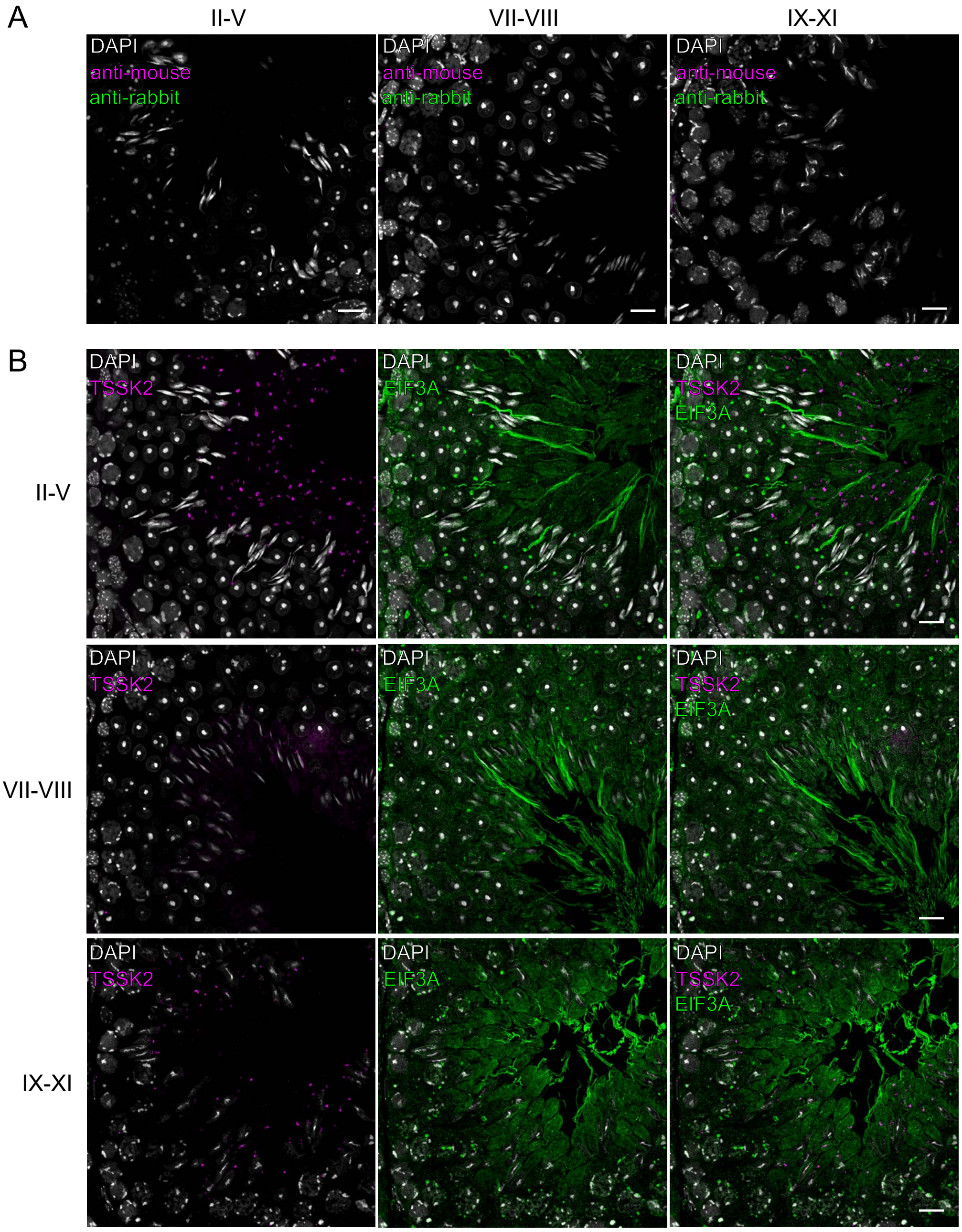

Supplementary Figure S1. Colocalization of EIF3A and TSSK2 in adult mouse testes. A) Negative control immunostaining (no primary antibody). B) Immunofluorescence of PFA-fixed paraffin-embedded adult testis sections with antibodies against TSSK2 (magenta) and EIF3A (green). DAPI stains nuclei (grey). Three stages (II-V, VII-VIII and IX-XI) of the seminiferous epithelial cycle are shown. Scale bar: 10 µm.

Supplementary Figure S2

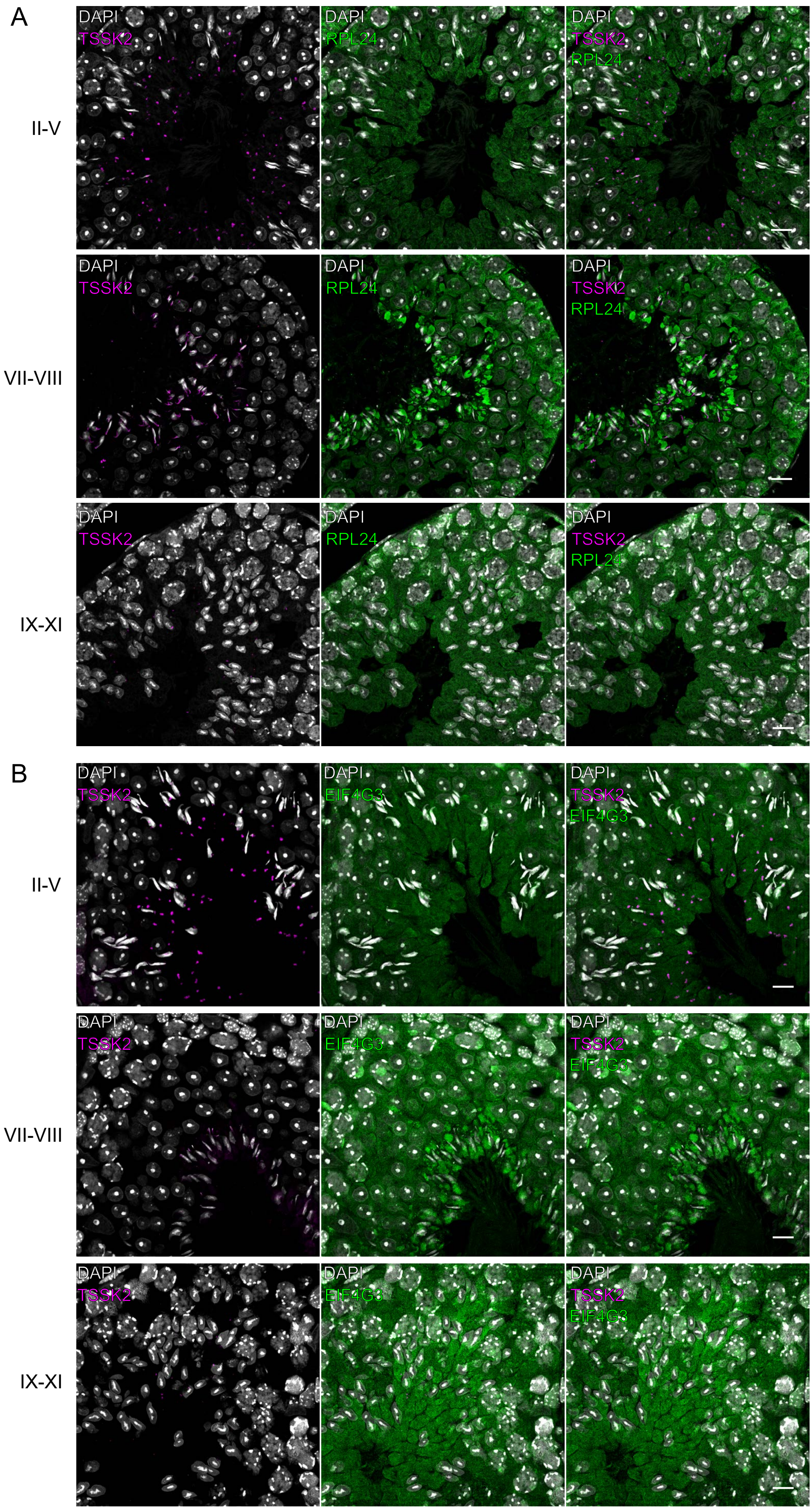

Supplementary Figure S2. Colocalization of RPL24 and EIF4G3 with TSSK2 in adult mouse testes. A) Immunofluorescence of PFA-fixed paraffin-embedded adult testis sections with antibodies against TSSK2 (magenta) and RPL24 (green). B) Immunofluorescence with antibodies against TSSK2 (magenta) and EIF4G3 (green). DAPI stains nuclei (grey). Three stages (II-V, VII-VIII and IX-XI) of the seminiferous epithelial cycle are shown. Scale bar: 10 µm.

Supplementary Figure S3

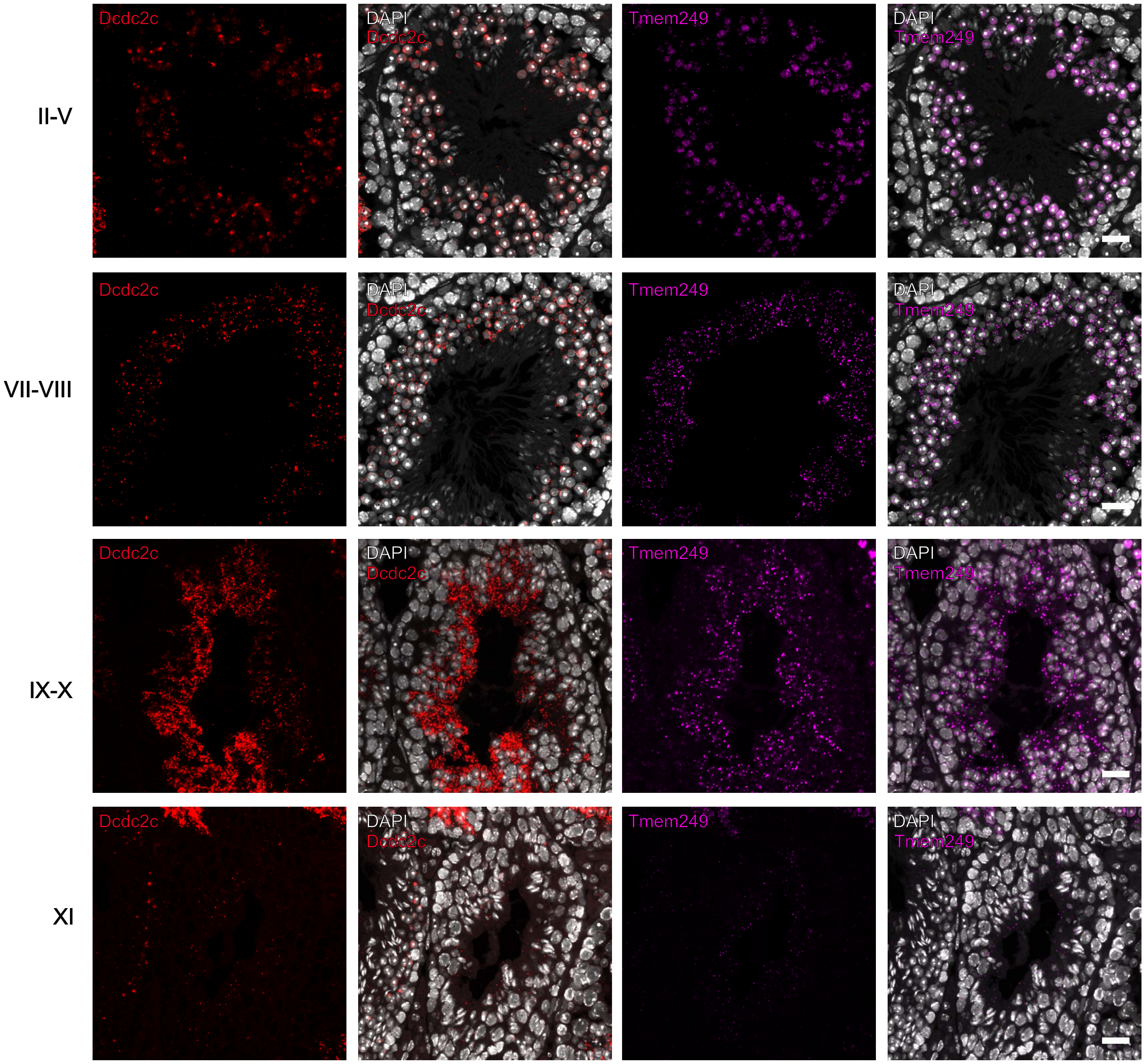

Supplementary Figure S3. Expression of *Dcdc2c* and *Tmem249* mRNAs in adult mouse testes. *In situ* hybridization of PFA-fixed paraffin-embedded testis sections using probes detecting *Dcdc2c* (red) and *Tmem249* (magenta) mRNAs. DAPI stains the nuclei. Representative images of the stages II-V, VII-VIII, IX-X and XI of the seminiferous epithelial cycle are shown. Scale bar: 20 μm.

Supplementary Figure S4

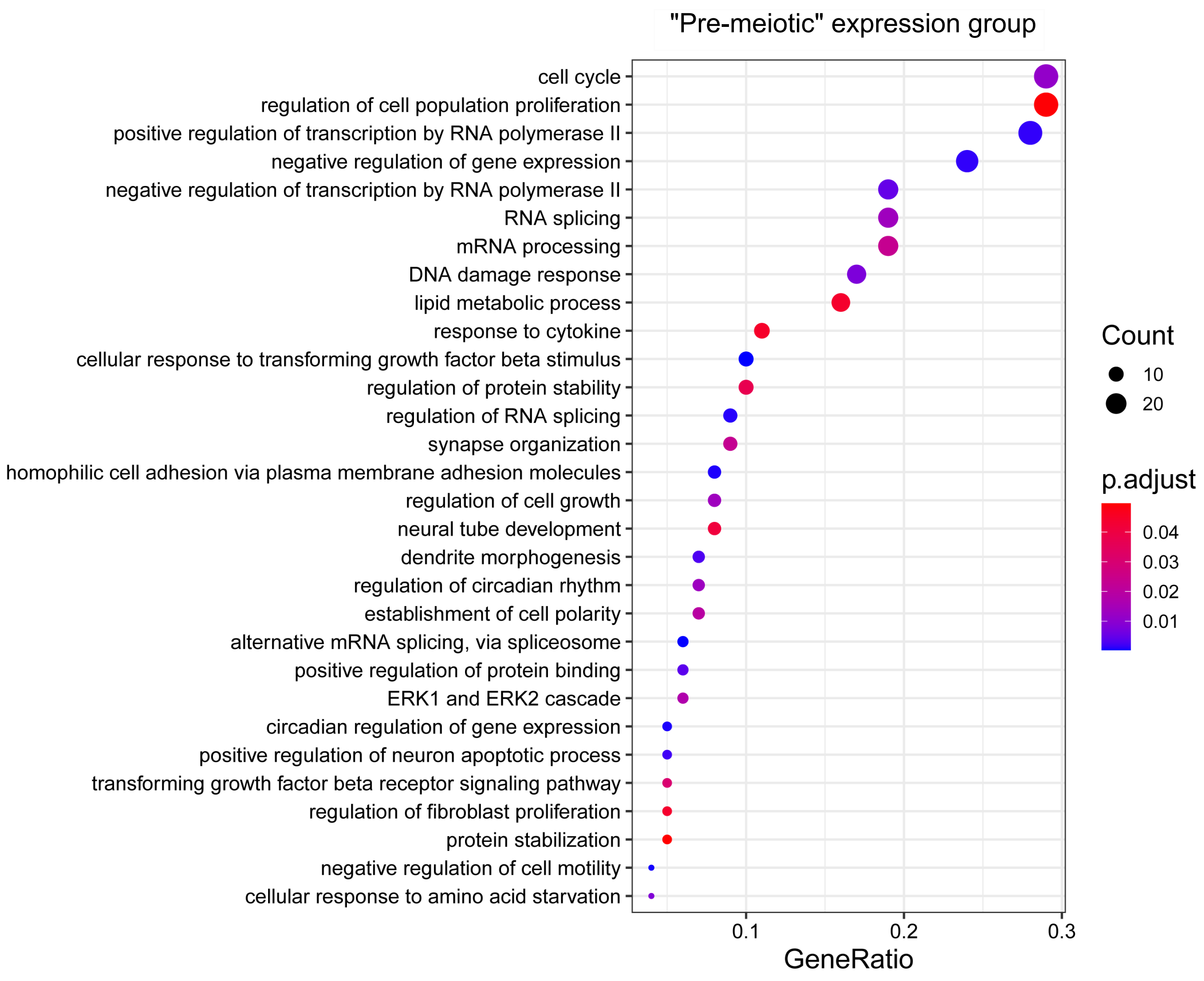

Supplementary Figure S4. GO enrichment analysis of the 247 TSSK2-associated mRNAs in the "Pre-meiotic" expression group. The first 30 Biological Processes were selected for visualization based on GeneRatio (the number of genes associated with the GO term divided by the total number of genes). p.adjust: adjusted p-values indicated by a gradient scale with red indicating the most significant values. Count: the number of genes associated to the GO term is illustrated by a dot with a proportional size according to the number of associated genes found in our gene list. See also Figure 5 and Supplementary Table S5.

Supplementary Figure S5

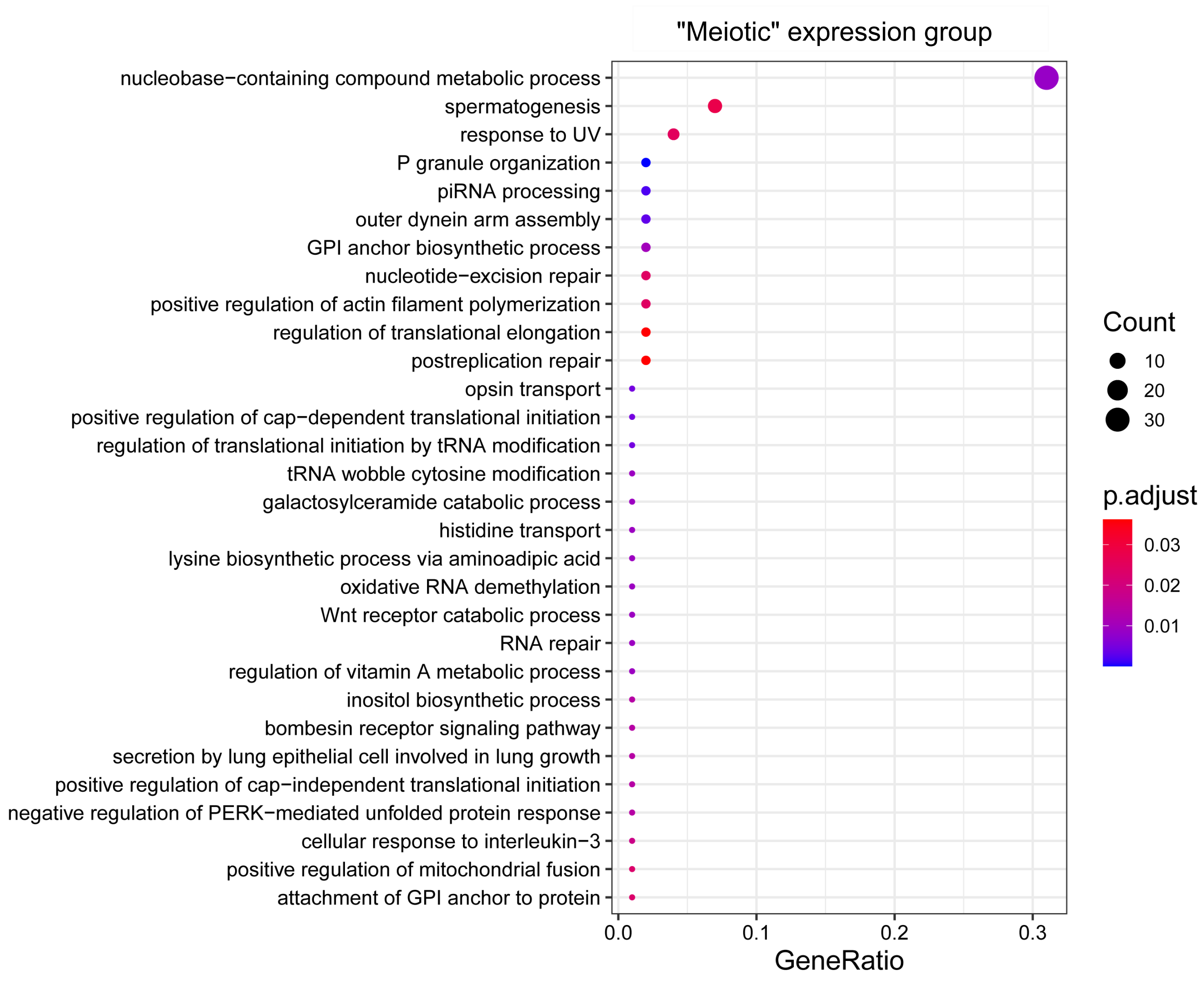

Supplementary Figure S5. GO enrichment analysis of the 100 TSSK2-associated mRNAs in the "Meiotic" expression group. The first 30 Biological Processes were selected for visualization based on GeneRatio (the number of genes associated with the GO term divided by the total number of genes). p.adjust: adjusted p-values indicated by a gradient scale with red indicating the most significant values. Count: the number of genes associated to the GO term is illustrated by a dot with a proportional size according to the number of associated genes found in our gene list. See also Figure 5 and Supplementary Table S6.

Supplementary Figure S6

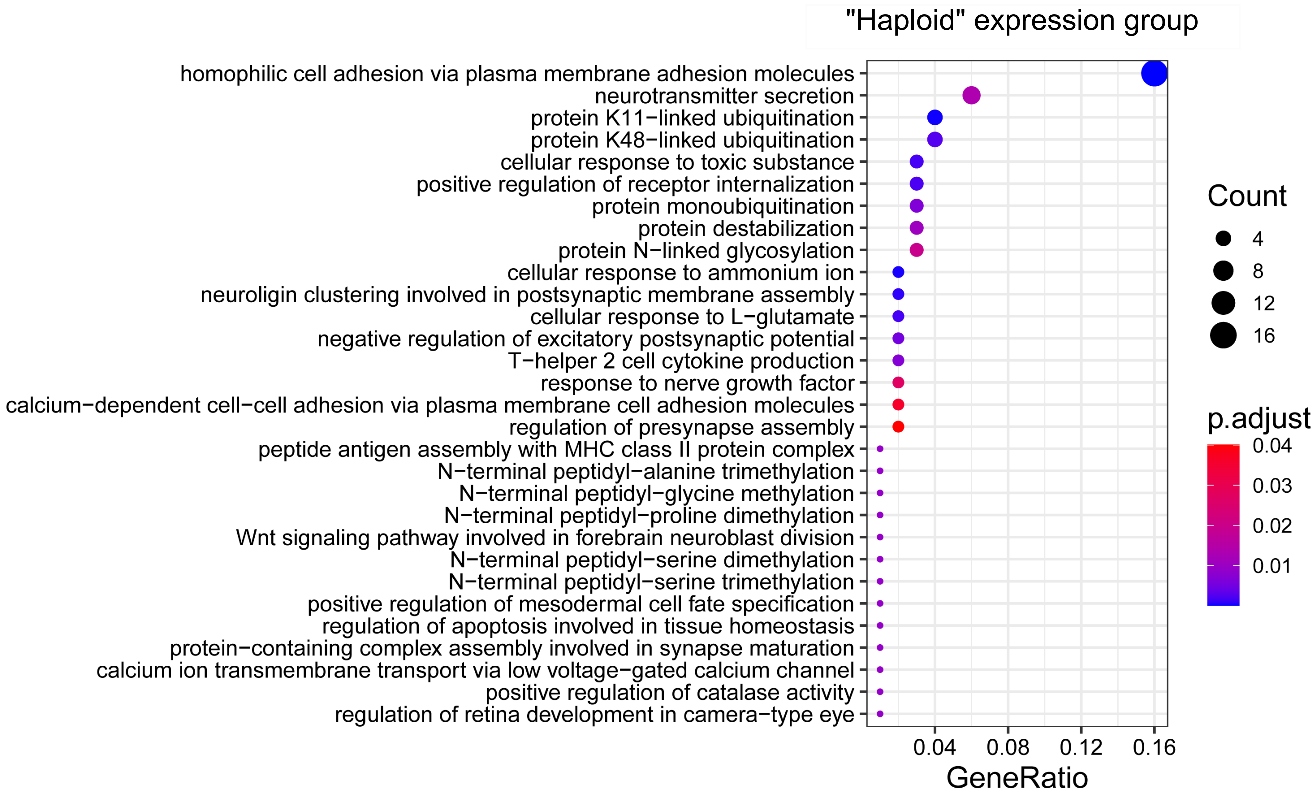

Supplementary Figure S6. GO enrichment analysis of the 201 TSSK2-associated mRNAs in the "Haploid" expression group. The first 30 Biological Processes were selected for visualization based on GeneRatio (the number of genes associated with the GO term divided by the total number of genes). p.adjust: adjusted p-values indicated by a gradient scale with red indicating the most significant values. Count: the number of genes associated to the GO term is illustrated by a dot with a proportional size according to the number of associated genes found in our gene list. See also Figure 5 and Supplementary Table S7.
